# The Book World of Medicine and Science

**Published:** 1896-05-02

**Authors:** 


### THE BOOK WORLD OF MEDICINE AND SCIENCE.

MANUAL OF GYNÆCOLOGY. By HENRY T. BYFORD, M.D. (Philadelphia: P. Blakiston, Son, and Co.; London: Kegan Paul, Trench, Trübner, and Co. 1895. Price 10s. 6d. net.)

The author has attempted a difficult task, and on the whole he has performed it well. His endeavour seems to have been to write a book which should teach to students those elements of gynæcology which they must know for the sake of passing examinations, and to practitioners those which are essential to enable them on the one hand to diagnose cases of pelvic disease, and on the other to take charge of their patients before and after the operations which may be done by specialists called in for the occasion; and yet to avoid entering into those details of operative work which are not required in general practice. No doubt some will be found ready to say that the result is a book on gynæcology with the gynæcology left out. But that is hardly a fair view of the matter. The various operations are mentioned, the instruments required and the preparation of the patient which is necessary are given, and the steps of the operations are shortly described, but not such details as would justify a man in undertaking the operations himself without consulting larger treatises. For the student, so far as examinations go, this is sufficient; for nine-tenths of the general practitioners it also is sufficient, and the rest can study larger works. Whatever people may say about and against the growth of specialism, it is a fact; and this book is but a type of many books which we fancy will in the future be written on many subjects; books, that is, written by specialists for general practitioners, and definitely accepting the limitations imposed by the fact that major operative work must in the future fall into the hands of the few. If we have any fault to find with the book before us it is that the author, having cut his readers off from the major operations, seems here and there to make peace with them by putting at their disposal a minor gynæcology of a rather fussy character. One very good point about the book is the great insistance upon cleanliness and antiseptic precautions which abound throughout its pages, and the very careful and minute instructions given in regard to all the proceedings to be taken in this regard both before and after all, even minor, operative work. It certainly is an interesting book, and for those who, while not aiming at being specialists, wish to receive full and exact information on such points in gynæcology as fall within their province as general practitioners, it will be of very great service.

TEXT-BOOK OF GENERAL PATHOLOGY AND PATHOLOGICAL ANATOMY. By RICHARD THOMA. Translated by Alexander Bruce, M.D. Vol. I., with 436 illustrations. Price 30s. (London: Adam and Charles Black, 1896.)

A translation into English of Richard Thoma's Pathology will be welcomed by pathologists in this country, since the Dorpat professor is recognised as a follower, or perhaps leader of the school that attacks the vital problems in pathology with the engines of chemistry and mechanics. Less of a philosophic speculator than many of his brethren in Germany, Thoma has made for hard facts and natural explanations of pathological phenomena, but although he has afforded many mechanical solutions to problems in development and teratology, he cannot be congratulated as having furthered our knowledge in perhaps the two chief pathological conundrums, namely, the etiology of cancers and the significance of heredity. Instances of predisposition to disease, and the pathological vagaries of development, afford perhaps richer fields for research in questions of heredity than do the physiological paths of biology, so that it is entirely unsatisfactory to learn that Professor Thoma sums up the position as regards the vexed question of the correlation between the variation of the organism and the external world, in the following sentence: "And should it at last appear that the processes by which the organism is built up necessarily involves (*sic*) the production of variations, we can only regard this as established with certainty, if these processes are seen to be the necessary result of the associated action of the forces of the external world which combine to form the organism."

With respect to the etiology of cancers, Professor Thoma is equally at a loss to refer their causation to any chemical or mechanical genesis—on this question his language is guarded, although reading between the lines it is not difficult to see that his fancies do not incline to support the parasitic origin. He sums up his creed in the following words: "We can therefore only make the general statement that mechanical, toxic, and infectious influences are of importance as regards the etiology of carcinoma, but we cannot as yet define this importance more exactly." The first volume, with its 600 pages, is devoted to questions of general pathology, with a considerable, but perhaps necessary, emphasis on the subject of bacteriology and diseases of parasitic origin. The chapter on malformations is one of the features of the book, with a profusion of illustrations, chiefly from original preparations collected by Professor Arthur Böttcher, his predecessor in the chair of pathology at Dorpat. Chapter VIII. deals with questions of circulatory disturbances, and here Professor Thoma is entirely on his own ground with mechanical explanations. Other chapters deal with disturbances of tissue nutrition, tumours, &c. The entire work is characterised by extreme care and accuracy, and the same may be said of the translation, which has been undertaken by Dr. Alexander Bruce. There is, however, a curious slip on the part of the author on page 12. In speaking of the influence of pathological causes on the germinal cells, Thoma remarks, "It is true that as yet we have not been able to demonstrate microscopically any changes occasioned by typhoid in the generative cells, but the same is true of the muscles, although it may be quite clear, from their excessive weakness, that they have been affected." On page 390 the author not only describes the degenerative changes in muscles during the course of typhoid, but he gives a most excellent illustration of the process.

